# Nurses’ Perspectives on Health Education and Health Literacy of Older Patients

**DOI:** 10.3390/ijerph17186455

**Published:** 2020-09-04

**Authors:** Min Young Kim, Seieun Oh

**Affiliations:** 1 College of Nursing, Jeju National University, Jeju 63243, Korea; musemy2@jejunu.ac.kr; 2 College of Nursing, Dankook University, Cheonan 31116, Korea

**Keywords:** older patients, older patients, patient education, health literacy, self-care

## Abstract

In the context of population aging, enhancing the health of older patients has become an urgent issue for public health. Health education and health literacy need to be further understood from the healthcare providers’ standpoint to increase older patients’ effective application of such information into their daily lives. We aimed to further understand nurses’ perspectives on the education of older patients and their health literacy, as nurses are one of the frontline providers interacting with older patients. In total, 16 nurses and nurse practitioners who had 5 or more years of clinical experience participated. Data were collected via face-to-face interviews and emails. Data analysis followed the thematic analysis suggested by Braun and Clarke. Five themes emerged from the analysis, as follows: attitudes that are hard to change; physical and cognitive functional barriers to understanding teaching materials; family caregivers—surrogate vs. gatekeeper; major contexts that moderate the elderly’s health literacy; and strategies to enhance teaching effectiveness and health literacy. These findings illustrate the conditions pertinent to communication with older adults from the patients’, providers’ and healthcare delivery viewpoints. Systemic assistance and interventions specialized for older patients and their healthcare providers need to be developed and tested to improve clinical practice and patient health literacy.

## 1. Introduction

Population aging, especially the rapid growth of the population aged 65 years or older, has resulted in many global issues and has challenged healthcare professionals to assist older people in maintaining optimal health status [1]. This has been particularly challenging because the number of older persons living with multiple health issues has increased as the population has aged, and therefore, older patients have been urged to manage their complex health conditions [2]. Thus, the improvement of self-care among older adults has become one of the priorities of healthcare providers nationally and internationally [1,3].

Previous research on health literacy and older people has clearly shown that older people have difficulties affecting their ability to self-care in addition to their complex health conditions. These include a high prevalence of cognitive impairment [4], including a significant decline of working memory and health literacy over time [5]. Limited health literacy has been associated with poor health outcomes [6]. Of the factors affecting the level of individual health literacy, education has been measured and shown to be positively related with health literacy among older patients [6].

As such, older persons with multiple conditions need to understand and follow complex instructions and absorb information to better maintain their health. Since health information is usually conveyed through patient education or counseling delivered by healthcare providers, further understanding of educational communication is imperative to assist older patients in performing better self-care. In this context, patient-centered communication has become the style of communication that healthcare professionals are supposed to pursue. Patient-centered healthcare providers deliver accurate and accessible information and provide the support necessary for patients’ self-care and health issue-related decision-making [1].

Ultimately, the aims of patient-centered communication are consistent with the concept of health literacy. While health literacy refers to an individual’s ability to understand and utilize health information so as to maintain health, in a broader sense, to promote health literacy it is critical to adjust the social environment in a manner pertinent to the individual utilization of health services. In particular, it is necessary to increase awareness of how healthcare providers and institutions deliver understandable and usable health information from the patient’s perspective [7]. Understanding healthcare providers’ attitudes toward health literacy is especially important because the concept of health literacy has not been fully established in Korea as an essential factor in providing the best care in hospital settings, even though the government has publicized patient-centered care as a goal of the Korean healthcare system since 2017 [8].

The healthcare paradigm shift to patient-centered care has set patient experience as the essential measure for quality of care, and two of the important evaluative dimensions of patient experience are communication and education [8]. Previous research on the outcomes of patient-centered care showed that studies frequently employed improving information, communication and education for patients [9]. Patient–physician communication has been found to have a significant impact on patient outcome and patients’ overall satisfaction with healthcare utilization [10]. Although patients’ satisfaction with nurses’ communication and care performance has also been found to have a significant positive impact on patients’ overall satisfaction [11], nurses’ communication and education directed towards older patients has been less studied compared to that of physicians.

Nurses’ communication patterns have been studied in the literature. Nurses perceived that they used more interactive skills (e.g., teach-back) than other health professionals [12], but they were perceived as engaging more in task-oriented communication than patient-centered communication due to their busy work from patients’ perspectives [3,13]. Inconsistency has also been found in the utilization and effectiveness of communication skills between nurses and patients [3]. However, the literature on nurses’ communication with patients has been limited to types of communication skills, patients’ recognition of such skills, and the quantitative comparison of the effectiveness of such skills between patients and nurses [3].

Accordingly, it is necessary to further understand healthcare providers’ perspectives concerning the education of older patients and their health literacy in relation to self-care. In clinical settings, nurses are frontline healthcare professionals responsible for providing direct care for older patients and meeting their educational needs [14]. Thus, this study aimed to elucidate nurses’ perspectives on the education and health literacy of older patients so as to provide better instructions and support for older patients’ self-care.

## 2. Methods

This study is part of a bigger project that aims to develop interventions to enhance the health literacy of older adults, by enhancing healthcare providers’ ability to interact with older adults in the context of patient education in tertiary general hospital settings, one of the mainstream healthcare systems in South Korea [8]. The project consists of interdependent studies to examine clinical communication patterns, their effectiveness, and barriers to both patients and healthcare providers’ use of health information, which will eventually be used to develop an intervention for healthcare providers.

### 2.1. Recruitment and Characteristics of the Participants

Ineligible participants for this study included registered nurses who had five or more years of clinical experience, as this was considered sufficient to have developed a certain degree of competency and strategies to deliver patient education on various topics. Since the context of this study was patient education for the self-care of older adults, we recruited study participants from departments related to various internal medicine subdivisions. Older adults frequently receive treatments at such departments, and education there is more likely to be about the long-term management of specific health conditions. Those working at departments irrelevant to providing direct nursing care for or interacting with patients, such as quality improvement divisions, were excluded. No other criteria were defined, as nurses interact with older patients in almost all hospital settings due to the rapid population aging in Korea.

Recruitment began in the hospital affiliated with the second author’s institution. The author introduced the study to the nursing department, and study fliers were distributed to general wards of internal medicine with the nursing manager’s permission. After the initial recruitment, a snowball technique was used to recruit the remaining participants. In total, 16 registered nurses and nurse practitioners (advanced practice registered nurses trained at the graduate level) participated in the study. They worked at four different general hospitals, with similar sizes (number of beds) and medical departments, in three Korean metropolitan cities. The mean age of the participants was 38.8 years (SD = 8.1, median 36.5, range 28–55). The average length of total clinical experience was 189.8 months (SD = 98.6, median 156.5), and the average time spent working at the current department was 106.5 months (SD = 80.7, median 82.5). Five nurse practitioners, seven registered nurses (RN) and four RN specialists participated in this study. Eight participants were working in general wards, three in hematology/oncology wards, four in cardiovascular/neurovascular centers, and one in a special unit. All participants were female. In their regular work, patient education comprised 50–70%, 20–30%, ≥90% and <10% of their time as reported by five, six, two and three.

### 2.2. Data Collection and Analysis

Data for the study were collected through face-to-face interviews and email communications. Participants were allowed to choose between the two methods because the distance of each participant from the researchers varied. Seven selected face-to-face interviews and the other nine participants chose to share their experiences through email. Face-to-face interviews ranged in duration from 25 to 50 min and took place at a participant-selected time and location. All interviews were audio-recorded with the participants’ permission and transcribed verbatim thereafter.

At the beginning of each interview, an informed consent procedure was administered. The purpose and background of the research were explained to the participants in detail since health literacy has not yet been emphasized in clinical settings in Korea. The same procedure was conducted via emails for those participants who chose the email method. A consent form was sent via email, with the introduction of the study scripted and consistently provided. Each participant was asked if she had any questions or concerns, but none was raised by any of the participants. After the introduction, seven interview questions were asked, and the same questions were used to collect data online. The questions were as follows: “How do you find working with older patients and their families?”; “Which aspects do you pay the most attention to in terms of educating patients and their families?”; “What difficulties are you facing when you educate or explain something to older patients?”; “What kinds of methods do you use to achieve your education goals when the patient does not understand well, and how do you employ them?”; “When you educate illiterate patients, if any, what are the most difficult issues if any?”; “What are the characteristics of limited health literacy?”; and “What do you think affects patients’ health literacy?”

Data analysis was conducted using thematic analysis, as suggested by Braun and Clarke [15]. All of the transcribed data and data written by participants were read multiple times to develop familiarity. During reading, emerging ideas or thoughts were written down. Pieces of the data that were thought to be important in relation to the research questions were initially coded, and such codes were then gathered based on relevance. Grouped codes were re-read to identify potential themes, each of which was reexamined with all of the relevant raw data for mutual exclusiveness and inter-relations. Thereafter, each theme was finalized and the characteristics and relationships among the themes were refined. The second author (SO) conducted the analysis, and the first author (MK) and another nurse researcher with expertise in gerontological nursing research and qualitative research experience reviewed the findings. No discrepancies were identified.

### 2.3. Establishing the Trustworthiness of the Study

Three methods were employed to establish the trustworthiness of the study: peer debriefing, maintenance of thorough records and materials for an audit trail, and thick (detailed) description of the study conditions. Through such methods, we tried to achieve research rigor for qualitative research as delineated by Lincoln and Guba [16]: credibility, transferability, dependability and confirmability. The first author and another nurse researcher served as peer debriefers to examine the understandability and agreeability of the findings. The entire research process and all of the relevant materials were systematically collected so that others could follow the data collection and analysis process. Detailed descriptions of the contextual information regarding each theme were provided so that readers could discern the transferability of the findings.

### 2.4. Ethical Considerations

This study was approved by the Human Subjects Committee at Dankook University Hospital (#2018–03-012). The participants’ human rights and privacy rights were protected throughout the study. Participants were allowed to choose their method of participation and to select the time and location of their interview. The informed consent procedure was consistently performed at the beginning of each online and offline interview session. We provided a thorough explanation of the study’s purpose, background and procedures, as well as benefits and potential risks of participation. The participant’s right to refuse to answer any question and to withdraw from the study at any time was also explained. Participants were encouraged to ask questions and express concerns. Data for the study were protected by separately storing signed consent forms, demographic information sheets and audio-recordings, or written answers, in locked cabinets and password-protected personal computers, and by assigning a code number to each participant in addition to the anonymization of any identifiable information.

## 3. Results

The findings of this study showed that participants perceived older patients to have multiple diverse conditions that are likely to hinder effective education, lower their health literacy, and thereby affect the efficacy of self-care practices. The participants focused on delivering accurate and detailed information on self-care practices for managing certain health conditions in their relatively unidirectional roles as providers. Time-regulated healthcare delivery in hospitals was the overall context under which nurses felt a large burden regarding educating or counseling older patients and their family caregivers. The five themes that emerged from the analysis are discussed in the following sections.

### 3.1. Theme 1: Attitudes that Are Hard to Change

Participants felt that working with older patients was difficult mainly due to a lack of understanding as to why older patients hold certain attitudes preventing them from engaging with health information. Such attitudes were described as generally having three characteristics: lack of interest in managing their health conditions, refusal to adapt and adjust to new health behaviors, and acceptance and maintenance of what they want only.

These three characteristics were perceived to be interrelated. Ignorance as to why they needed to change their behaviors was one of the underlying ideas, reinforced by resistance to new environments or new lifestyles for health management. Lack of interest in life modification was often accompanied by an absence of responses or absentminded responses during education. It was difficult for the nurse or nurse practitioners to determine if they understood the content, and the extent of their comprehension.

*Well, I think older patients seem to have resistance to new circumstances and new things. Some patients insist that they have the same prescription no matter what, even if drugs need to be changed or new tests are needed. Doctors and nurses explain it and persuade them over and over again, but they do not even listen. Sometimes, they say ‘I understood it all’, but after a while, it later turns out that they did not understand the content at all.*

(HCP_01)

*When I teach patients who take oral chemo drugs how to use gloves and precautions regarding bathroom use, they then say, “I live alone, so it’s okay [not to follow the instructions]”, “I can take the drugs without touching them with my bare hands”, “Do I really need to do things like this?”…So there’s no cooperation in many cases. When older couples or older patients living without anyone else say, “I am going to die anyway, so should I really follow these rules?”, then I am like, speechless. This is something that I still have a hard time with.*

(HCP_03)

Some older patients exhibited a proactive attitude of engaging in healthy, recommended behaviors and successful self-care. However, it was hard to identify what caused such a difference in attitudes toward self-care among older patients. Understanding the reasons for certain attitudes might be a starting point for enhancing older patients’ health literacy, but they were practically impossible to determine due to the limited time available to educate each person in clinical settings.

### 3.2. Theme 2: Physical and Cognitive Functional Barriers to Understanding Teaching Materials

A short attention span, a decreased ability to understand and memory problems or loss were significant functional limitations that hindered effective education and frustrated or discouraged the participants. Due to these limitations, educational sessions for older adults should be brief, taught slowly, and repeated as many times as necessary, which may demand more time and energy from healthcare staff. Although the nurses put much more time and effort into the education of elderly patients, the patients’ misinterpretation of the given instructions or even total obliviousness to the fact that they had received the education was commonly observed in follow-ups. The accumulation of such incidences over time, especially given the efforts and time they put in, seemed to lead to participants’ negative generalizations of older patients. Patients’ denial of receipt of education despite having received the education made the participants feel like it was not worth investing their time and raised concerns that coworkers might view them as negligent.

*It could happen to (younger) adult patients though, but… (…) There was a case that I educated and consulted an older patient who did thank me at the end of the session, with a smile. Later, his daughter called me and yelled at me because the patient told her the very opposite of what I had explained to him. Who could I blame? So, I told her that it seemed I did not explain it to him well enough and then explained it again. When I encounter such incidences, I feel like I’m missing something and I get hurt so much.*

(HCP_02)

Hearing difficulties and poor eyesight are common problems among elderly patients that hinder their communication with nurses. Ready-made educational materials for the general population are not suitable for elderly patients in terms of font sizes, terminology used, and the range of information included. The lack of resources designed exclusively for older patients requires participants to spend extra time and effort adjusting these materials to convey concrete, essential and succinct take-home messages so that older patients can use them at home to maintain their health and manage their disease.

*Settings, materials, equipment and tools are not specialized for elderly patients, so it is hard to use anything as it is. Because the font size of written instructions is fixed, I need to highlight or re-write key content in larger letters so that the patient can easily recognize it later.*

(HCP_09)

### 3.3. Theme 3: Family Caregivers: Surrogate vs. Gatekeeper

Experiences with elderly patients who were concerned because of a misunderstanding, or total obliviousness, of instructions given to the patient naturally led to the participants asking family caregivers to be present and participate in the patient education or counseling session. Some participants were more likely to focus on educating the family caregiver rather than the patient. This practice was based on their previous experiences in which they found teaching the family caregiver to be a more effective means of instruction. In such cases, the caregiver was usually an adult child of the patient. However, this method has inevitable limitations, especially when they do not live together. More importantly, because they are not the patients themselves, it is difficult to manage risk factors thoroughly and systemically. In addition, this approach cannot guarantee the patient’s proper understanding of and compliance with the recommended medical regimen.

When the caregiver was an older adult as well, usually a spouse, any available adult child was also requested to attend so as to ensure that the information provided was received correctly. In such circumstances, attitudes toward caring for the patients (i.e., proactive or reluctant) and the actual capacity of support from the family caregiver determine the depth and range of patient education at the scene.

While the cases mentioned above were about family caregivers as supporters, reversed power relationships between the patient and the family caregivers were also often observed. Older patients were the messengers between the doctors and the family members, while the decision-making authority was held by the family caregiver, not the patient. However, both the patient and the nurse participants seemed to naturally accept such situations without concern.

*Older patients usually come with their adult children, so when they need to make a decision regarding treatment, they discuss it with their children. It is a good thing, but sometimes it makes my job very hard…So it requires sufficient explanation for the children as well. If the child caregiver refuses to allow his or her parent to receive certain treatments, then it is very difficult. Once, a son of my patient told me, “I am going to report you to the police.”*

(HCP_12)

### 3.4. Theme 4: Major Contexts that Moderate Elderly People’s Health Literacy

Conditions that might affect adherence to medical recommendations or self-care were perceived by participants as contexts under which the level of health literacy of an older patient may vary. Such conditions include living alone or with an elderly spouse, financial vulnerability, lower educational level, lack of supportive systems (e.g., inactive care involvement of nonresident family caregivers), and easy access to a plethora of unproven health information from the media.

Participants also perceived these conditions as barriers that prevent older patients from obtaining information generally known to the public, or from prioritizing their health. It seems that this is where gaps of conceptualization between healthcare providers and older patients may begin. Some participants pointed out that all of the disadvantaging conditions described above play a significant role together in the loss of decision-making capability among vulnerable older patients. In such cases, explanations need to be more concrete and detailed so that the patient can understand what the nurse is trying to convey. One participant’s comment exemplified this context:

*Patients who have low health literacy are usually socially vulnerable. How can I put this …Some things that ordinary people see as “common sense”, are sometimes not known by those with low health literacy. For example, when I say “Do not take any home remedies”, they say, “I do not do it”, but after asking for details, I find that patients don’t consider vegetable or fruit extracts are not home remedies. They are just waters. In such circumstances, when educating vulnerable older patients, explanations need to be very concrete and based on real things. Another major concern is that the mass media releases a lot of unproven information on health. Because those older patients have a limited capability to judge the truth, they unconditionally accept and apply it by themselves, ultimately ruining their health.*

(HCP_06)

Time pressure for both healthcare providers and patients was a fundamental aspect of the clinical context, preventing sufficient education or counseling to resolve such conceptual gaps from taking place. Thus, patients were not allowed sufficient time to digest what was taught and enhance their health literacy.

### 3.5. Theme 5: Strategies to Enhance Teaching Effectiveness and Health Literacy

With so many functional and psychological limitations, participants utilized various means to deliver information pertinent to managing particular health conditions. The focus of elderly education or counseling varied (e.g., medication compliance, risk factor management or symptom management) depending on the educator’s working department. The majority of the educational content is complex and requires behavioral changes and relevant decision-making, rather than simple fixed actions.

The study participants employed invisible but essential processes, such as adjusting the depth and breadth of information conveyed to the patients. Such adjustments were made based on the participant’s subjective judgment of the patient’s comprehension level and capacity to follow the given instructions in their everyday life. While they can sometimes obtain such information from coworkers in other departments, nurses usually perform their own investigation based on their experiences and expertise.

*I think it is really important to figure out how much the patient can understand and accept [the content] before and during the education session. Further, how much support the family caregiver can contribute is very important as well. If a patient shows a low level of understanding and limited capability to self-care, then I would explain and emphasize only the very fundamental and “must-do” things, like “take your medication at the right time, do not skip meals, check for symptoms of and properly respond to hypoglycemia.”. (…) If a patient has high compliance and can understand, then I would extend the instructions to include HbA1C interpretation, its target level, and other test results needed to manage.*

(HCP_07)

Other strategies frequently used by the participants included highlighting and summarizing key information only, repetition of explanations, questioning of the patient to confirm that the patient understood the content, writing to-do lists at the end of the session, drawing pictures, and utilizing real-life objects and examples, and actual pills or equipment.

*I usually give drawings or pictures. In terms of cautions, I write them on a separate sheet and tell the patient to tape it on the wall so they do not forget them. If it does not work for the patient, then I try to understand his/her life patterns and tell him or her the do’s and don’ts out of them, and that way usually works. For example, if the patient wants to eat carbs like potatoes or sweet potatoes for snacks after meals, then I tell them “Please don’t do that”. If you still want to eat them, have them as a meal, not for a snack.*

(HCP_11)

There were few encounters with illiterate elderly patients. However, during such encounters, reliance on and education of family caregivers became more crucial, since all of the written materials or handwritten summaries were useless for those patients. Family caregivers were requested to check on the patient and contact him or her more frequently. In addition to educating family caregivers, participants frequently used verbal explanations, pictures and real-life materials for such patients.

## 4. Discussion

This study attempted to understand the patterns of and barriers to effective communication, as perceived by nurse clinicians in the context of patient education for the self-care of older adults from the perspective of health literacy. The findings of this study showed that multiple factors are involved in patient education, from older patients’ physio-psychological conditions to time-regulated healthcare services.

Participants reported older patients’ resistant attitudes to be the most difficult issue experienced. Seemingly, the nurses’ lack of understanding of why older patients hold such attitudes needs to be resolved. Those who express rejection of or resistance to new recommendations can be easily labeled as “non-compliant.” However, among them, a significant percentage have undetected decreased or impaired cognitive function, and thus limited health literacy [4], and healthcare professionals are unable to recognize over 40% of such problems [17]. Moreover, older patients with limited health literacy are not likely to realize when they do not comprehend health information [18]. Additionally, other unspoken practical problems might prevent them from understanding or following recommendations. As such, steps to identify older patients’ specific issues regarding health literacy need to be established within the service usage trajectory, and patient education should be designed to address each patient’s particular issues [19].

More importantly, participants’ views on patient education are still geared toward the provider’s compliance perspective, assuming that the patient’s role is as a listener rather than as a partner. This conflicts with what “patient-centered communication” emphasizes: building partnership between healthcare providers and patients rather than a hierarchical relationship [1]. In the context of partnership, healthcare providers should seek and respect patients’ opinions and choices, including reasons not to integrate the recommendations and treatment options. Patient education, subsequently, needs to focus on sustainable action plans that a patient can perform daily as much as possible. As Marcus [20] suggested, the ideal roles of both healthcare providers and patients in the context of patient education and communication should be studied further.

Healthcare professionals must pay more attention to older patients’ internalizations of social images of aging and the aged. Some of the participants’ descriptions of older patients’ attitudes reflected social images of aging and aged persons (e.g., it is not worth it for older adults to try to achieve a better life). Consequently, such attitudes were more likely to be linked with undesirable health outcomes. Images about aging internalized by the elderly can have a significant impact on the functional and psychological health of elderly clients [21,22,23,24]. Thus, from a public health standpoint, it is necessary to employ orchestrated efforts in order to change the negative images of older people at both the social and individual levels.

Older patients’ over-reliance on family caregivers in terms of decision-making can be understood from an age-related cultural standpoint. In traditional Korean culture, under which most of the current older population was raised and socialized, older people are supposed to lean on their children, and older persons are supposed to be weak, ignorant and dependent [25]. For adult children, taking good care of aged parents can be considered filial piety [26]. Within such a culture, it may be natural for both patients and healthcare providers to accept their dependence in decision-making. Although living fully independently from their children is not feasible, interventions to improve older patients’ independence as much as possible are worth developing and testing.

The negative experiences of the participants have implications for preventing ageism in clinical settings. The negative influence of ageism on the health of older patients has been well established in the literature [22]. Thus, nurse educators’ discriminative attitudes due to ageism can reduce the quality of communication with older patients, and, more importantly, their health literacy. Although participants’ current ways of communicating or interacting with older patients do not correspond with obvious age-discriminative behaviors, repetitive exposure to negative experiences with older patients might build a fixed general negative impression of older patient groups. Since gerontology-specialized healthcare providers are not always available in every clinical setting, there is an urgent need to develop in-service training on communication with older patients and ageism prevention for employees, and to provide necessary consultation on how to deal with such negative encounters.

The gaps in conceptualization between the nurses and patients found in the study need to be considered in any educational context, especially for patients with limited health literacy. Such gaps can be found in conceptualizing or interpreting even ordinary words, not just professional jargon [19]. As such, the level of health literacy depends on the content rather than the personal literacy level; limited health literacy cannot be improved by merely simplifying the information or format [27]. Thus, thorough discussions regarding key concepts and vocabularies used during patient education are urgently needed to confirm that each party is working at the same level.

Strategies used by the participants resonate with the skills recommended for limited health literacy and frequently used by healthcare professionals [1,3,28], namely using simple language, narrowing down the range of education, and speaking slowly. While confirmation of comprehension was one of the critical components of education reported by the participants, methods of confirmation are somewhat limited to asking questions, rather than using interactive skills, such as teaching-back, which is also consistent with findings from previous studies [3]. It is necessary to evaluate the effectiveness of the methods or tools used by nurse educators (e.g., highlighting key information, providing a separate summary sheet) from the patient’s perspective, and to develop and test effective means to confirm the patient’s comprehension of the teaching material. Integrating the learning styles of individual patients can be worth trying in order to enhance the effectiveness of patient education, and subsequently their health literacy [29].

Fundamentally, training in communication and health literacy for healthcare providers needs to be applied regularly. Previous research shows that healthcare professionals who took any communication course or were aware of the concept of health literacy employed more skills to enhance patients’ health literacy. As Pitt and colleagues suggested [30], systemic efforts, such as regular audits or feedback on provider–patient communication, can help increase awareness of the provider’s performance, and thereby enhance the quality and effectiveness of health communication.

Healthcare practitioners must pay more attention to the heavy reliance on family caregivers in teaching patients. Older adults with multiple health conditions tend to rely on family caregivers [2]. Involving a family member in discussions has been recommended for older patients with limited health literacy [12,31]. However, the findings suggest that the nurses actually educate family caregivers rather than having them support the patient. Nurses do not always expect their full engagement in patient care. Expecting them to supervise the patient or assist with his or her self-care could place a heavy burden on family members, and thus, it has certain limitations. Instead of placing the entire burden on family caregivers, it would be more practical for a nurse educator to ask them to help to build sustainable self-care plans for the patient and give them a few detailed tasks, the feasibility of which can be confirmed with the family members.

It is imperative to build care continuity between tertiary hospitals, where older patients receive instructions for the self-care of certain health conditions, and community resources so as to distribute the responsibility for monitoring and assisting with older patients’ self-care. Since patient education in clinical settings faces time pressures globally, it may be impractical to perform all of the patient-centered procedures, including teaching-back or in-depth discussions on integrating the medical recommendations into daily life. Providing continuous care will help older patients to discern useful information out of the vast majority of health information released by the mass media as well.

Lastly, effective communication and education for patients can help them become health literate, but cannot guarantee behavioral changes. Future research, possibly with longitudinal designs, should pay close attention to how patient education received from hospitals does or does not help patients adopt the recommended behaviors.

A few characteristics of the current study need to be resolved in future research. The participants of this study were all female, and thus, male nurses’ experiences and perspectives were not included. As experiences and perceptions of communication with patients can differ by gender [3], diverse perspectives need to be included in order to further understand communication contexts for better practice. The working departments of the participants included in this study were concentrated on internal medicine; other departments require different types of self-care, and thus, future research needs to include more diverse educational situations. Although the literature on health literacy and communication in clinical settings has studied the perspectives of healthcare professionals or patients, similar to the current study, there is still a need to conduct research using interaction analyses of the actual conversations and interactions, so as to triangulate what is happening with what is being perceived in clinical settings.

## 5. Conclusions

The results of this study have shed light on the aspects of communication with and education of older patients that healthcare providers need to improve. Older patients’ functional limitations and internalized images of aging and the elderly have prevented them from actively performing healthy, recommended behaviors. Lack of a systemic approach to measure and utilize health literacy information, lack of partnership formation with and assistance specialized for older patients, and time-regulated healthcare delivery, have also built barriers that block both effective patient education and the enhancement of the health literacy of older patients. Motivations for self-care and the encouragement of older patients’ independence are much needed topics for future research. Lastly, assistance and interventions for healthcare professionals who have multiple negative experiences will prevent the amplification of ageism in clinical practice.
